# MiR-7-5p-mediated downregulation of PARP1 impacts DNA homologous recombination repair and resistance to doxorubicin in small cell lung cancer

**DOI:** 10.1186/s12885-019-5798-7

**Published:** 2019-06-18

**Authors:** Jinzhi Lai, Hainan Yang, Yanyang Zhu, Mei Ruan, Yayu Huang, Qiuyu Zhang

**Affiliations:** 1 Department of Oncology, The Second Affiliated Hospital, Fujian Medical University, Quanzhou, China; 2 Institute of Immunotherapy, Fujian Medical University, Fuzhou, 350108 Fujian China; 3 Department of Ultrasound, The Second Affiliated Hospital, Fujian Medical University, Quanzhou, China

**Keywords:** Small cell lung cancer, MiR-7-5p, Chemo-resistance, Doxorubicin, Poly ADP-ribose polymerase 1, Homologous recombination

## Abstract

**Background:**

Chemo-resistance is one of the major challenges in the therapy of small cell lung cancer (SCLC). Multiple mechanisms are thought to be involved in chemo-resistance during SCLC treatment, but unfortunately, these mechanisms have not been well elucidated. Herein, we investigated the role of miRNA in the resistance of SCLC cells to doxorubicin (Dox).

**Methods:**

MiRNA microarray analysis revealed that several miRNAs, including miR-7-5p, were specifically decreased in Dox-resistant SCLC cells (H69AR) compared to parental cells (H69). The expression level of miR-7-5p was confirmed by qRT-PCR in Dox-resistant cells (H69AR and H446AR cells) and their parental cells. Bioinformatic analysis indicated that poly ADP-ribose polymerase 1 (PARP1) is a direct target of miR-7-5p. The binding sites of miR-7-5p in the PARP1 3′ UTR were verified by luciferase reporter and Western blot assays. To investigate the role of miR-7-5p in the chemo-resistance of SCLC cells to doxorubicin, mimic or inhibitor of miR-7-5p was transfected into SCLC cells, and the effect of miR-7-5p on homologous recombination (HR) repair was analyzed by HR reporter assays. Furthermore, the expression of HR repair factors (Rad51 and BRCA1) induced by doxorubicin was detected by Western blot and immunofluorescent staining in H446AR cells transfected with miR-7-5p mimic.

**Results:**

The expression level of miR-7-5p was remarkably reduced (4-fold) in Dox-resistant SCLC cells (H69AR and H446AR cells) compared with that in parental cells (H69 and H446 cells). Poly ADP-ribose polymerase 1 (PARP1) is a direct target of miR-7-5p, and PARP1 expression was downregulated by miR-7-5p. MiR-7-5p impeded Dox-induced HR repair by inhibiting the expression of HR repair factors (Rad51 and BRCA1) that resulted in resensitizing SCLC cells to doxorubicin.

**Conclusions:**

Our findings provide evidence that miR-7-5p targets PARP1 to exert its suppressive effects on HR repair, indicating that the alteration of the expression of miR-7-5p may be a promising strategy for overcoming chemo-resistance in SCLC therapy.

**Electronic supplementary material:**

The online version of this article (10.1186/s12885-019-5798-7) contains supplementary material, which is available to authorized users.

## Background

Lung cancer is the leading cause of cancer death worldwide, and small cell lung cancer (SCLC) accounts for approximately 15 to 20% of all lung cancers [1]. The standard chemotherapy regimen for SCLC uses topoisomerase inhibitors in combination with cisplatin. SCLC is characterized by the rapid development of resistance to drugs even when there is an initial response [2]. Acquired chemo-resistance is considered the major drawback of current chemotherapeutic regimens, but the molecular details have not been completely elucidated. Hence, there is an urgent need to identify the underlying mechanisms of chemo-resistance and to explore effective strategies to overcome resistance.

DNA-damaging agents are the most widely used chemotherapeutic drugs [3]. DNA-damaging agents, such as doxorubicin (Adriamycin, Dox), prevent cell division and lead to cell death by inhibiting the religation of DNA strands in double-strand breaks (DSBs) [4]. However, cancer cells may acquire chemo-resistance by altering the cell survival signaling pathway and repairing the DNA damage [5]. The DNA damage response (DDR) is a molecular mechanism that cancer cells have exploited to activate DNA repair pathways and prevent DNA damage-induced cell death [6].

Among these DNA repair pathways, homologous recombination (HR) is one of the key pathways for the repair of DSBs [7]. A variety of DDR proteins are involved in the regulation of HR, resulting in cancer drug resistance [8]. The expression of DNA repair proteins has recently been reported to be regulated by miRNAs (microRNAs). MiRNAs are small, 20–23-nucleotide noncoding RNAs that are known typically to suppress gene expression by binding to complementary sequences in the 3′-untranslated regions (3′ UTR) of target genes [9]. Recently, the association between miRNA expression and chemo-resistance in cancer was noted [10]. Some studies demonstrated that changes in the expression levels of miRNAs may be involved in tumor cell resistance to chemotherapy by regulating the efficacy of DNA damage repair processes [11, 12]. Nevertheless, the molecular mechanisms underlying the role of miRNAs in chemo-resistance are not yet fully understood in SCLC.

In this study, we conducted an array-based analysis of microRNA expression patterns by comparing Dox-resistant H69AR cells and their parental cells. Among 62 differentially expressed miRNAs, miRNA-7-5p (microR-7-5p) exhibited a remarkably decreased level in H69AR cells. MiR-7-5p has previously been reported to be a tumor suppressor in multiple cancer types and inhibits growth and invasion [13, 14]. However, to date, no available studies have conclusively demonstrated the association between miR-7-5p and DNA repair in SCLC chemo-resistance. Here, we revealed that miR-7-5p-facilitated HR repair contributes to chemo-resistance in SCLC cells by targeting poly ADP-ribose polymerase 1 (PARP1). Our findings provide an understanding of the function of miR-7-5p in the resistance of SCLC to chemotherapy.

## Methods

### Cell lines and cell culture

A human SCLC cell line (H69) and a Dox-resistant cell line (H69AR) were purchased from the American Type Culture Collection (Manassas, USA). The H446AR cell line is a Dox-resistant SCLC cell line that was derived from H446 in our lab. These cell lines were maintained in RPMI 1640 supplemented with 10% fetal bovine serum (Sigma-Aldrich, USA). The H69AR and H446AR cell lines were challenged monthly to maintain resistance to doxorubicin (MedChemExpress, USA). The Dox-resistant cells were maintained in drug-free medium for at least 2 weeks before any experiments.

### MiRNA microarray analysis

Total RNA containing miRNA was extracted from H69 and H69AR cells with Trizol reagent (Invitrogen, USA) and purified with the mirVana miRNA Isolation Kit (Ambion, USA) according to the manufacturer’s protocol. MicroRNA profiling was performed using an Agilent miRNA array. Briefly, miRNAs were labeled using the Agilent miRNA labeling reagent. Total RNA (200 ng) was dephosphorylated and ligated with pCp-Cy3, and the labeled RNA was purified and hybridized to miRNA arrays. Images were scanned with the Agilent microarray scanner, gridded, and analyzed using Agilent Feature Extraction Software version 10.10.

### MiRNA transfection

The miR-7-5p mimic and inhibitor were synthesized by Sangon Biotech (Shanghai, China). The sequences of the synthetic oligonucleotides were as follows: miR-7-5p mimic (miR-mimic) sense 5′-UGGAAGACUAGUGAUUUUGUUGUU-3′, antisense 5′-CAACAAAAUCACUAGUCUUCCAUU-3′; miR-7-5p mimic NC (NC-mimic) sense 5′-UUCUCCGAACGUGUCACGUTT-3′, antisense 5′-ACGUGACACGUUCGGAGAATT-3′; miR-7-5p inhibitor (miR-inhibitor) 5′-AACAACAAAAUCACUAGUCUUCCA-3′; miR-7-5p inhibitor NC (NC-inhibitor) 5′-CAGUACUUUUGUGUAGUACAA-3′. These synthetic oligonucleotides were transiently transfected into SCLC cells by Lipofectamine 3000 (Thermo Fisher Scientific, USA) and Opti-MEM (Invitrogen, USA) according to the manufacturer’s protocol.

### MiRNA isolation and quantitative real-time PCR

Total RNA was extracted from cells and reverse-transcribed into cDNA using the miRNA First Strand cDNA Synthesis Kit (Sangon Biotech, China) according to the manufacturer’s protocol. Quantitative real-time PCR (qRT-PCR) was performed using a SYBR® Prime Script™ RT-PCR Kit (Invitrogen, USA). The qRT-PCR primers were synthesized by Sangon Biotech. The forward primer for miR-7-5p was (5′-GCGCTGGAAGACTAGTGATTTTGTTGTT-3′) and the reverse primer for U6 was (5′-CTCGCTTCGGCAGCACA-3′). The universal reverse primer was (5′-AACGCTTCACGAATTTGCGT-3′). The relative expression of miRNA was calculated by normalization against that of the U6 small nuclear RNA.

### Western blot analysis

The total protein from SCLC cells was extracted using RIPA lysis buffer. The protein lysates were separated by 10% SDS-PAGE and electrophoretically transferred to PVDF membranes (Millipore, USA). The membranes were incubated with primary antibodies at 4 °C overnight, followed by incubation with secondary antibodies. The signals were detected using an ECL system (Thermo Fisher Scientific, USA). The intensity of the protein fragments was quantified with Image Lab software (Version 5.2.1 build 11, Bio-Rad). Anti-PARP1 antibody (Cell Signaling Technology, USA), anti-BRCA1 antibody (Santa Cruz, USA), anti-Rad51 antibody (Abcam, USA), anti-GAPDH and anti-β-tubulin (Cell Signaling Technology, USA) were used as the primary antibodies for the detection of specific proteins.

### Cell counting kit-8 (CCK-8) assay

SCLC cells were plated in 96-well plates at 5 × 10^3^ cells per well and treated with doxorubicin for 24 h. The absorbance at 450 nm was measured after incubation with 10 μl CCK-8 reagent (Dojindo, Japan) for 4 h. The obtained values were used to calculate the IC50 using untreated cells as a control. The assay was conducted in three replicate wells for each sample, and three parallel experiments were performed.

### Dual-luciferase reporter assay

To determine the target sites of miR-7-5p on the 3′ UTR of PARP1 mRNA, HEK293 cells were seeded in 24-well plates and cotransfected with wild-type or mutated (one point mutation) PARP1 3′ UTR reporter plasmids for 24 h using Lipofectamine 3000. Then, the cells were transfected with miR-7-5p mimic or inhibitor for another 24 h. The cells were harvested for luciferase activity assays that were performed using the Dual-Luciferase Reporter Assay System (Promega, Germany).

### HR reporter assay

The HR repair assay was carried out using the DR-GFP reporter system developed by Maria Jasin. Cells were seeded in 24-well plates and cotransfected with pDRGFP (Addgene plasmid 26,475) and pCBASceI (Addgene plasmid 26,477) by Lipofectamine 3000. The percentage of GFP-positive cells determined by flow cytometry provided a quantitative measure of HR efficiency.

### Immunofluorescent staining

H446R cells were transfected with miR-7-5p mimic or control miRNA for 24 h, and then cells were treated with 25 μg/ml doxorubicin for another 24 h. The cells were fixed and stained with mouse anti-BRCA1 (1:100 dilution; Santa Cruz, USA) and rabbit anti-Rad51 (1:66.67 dilution; Santa Cruz, USA). Goat anti-mouse IgG-Alexa Fluor® 594 or chicken anti-rabbit IgG-Alexa Fluor® 488 (1:400 dilution; Invitrogen, USA) was used as the secondary antibody. Photos were taken with a fluorescence microscope (Life Technologies, USA) at a magnification of 200 × .

### Colony formation assays

Cells were seeded into 60 mm dishes at 200 cells per dish and treated with ABT-888, Dox or a combination of these for 24 h, and then medium was replaced with fresh medium. The cells were stained using Giemsa stain after 14 d of incubation, and colonies containing > 50 cells were counted. The mean value ± SD for three independent experiments was determined.

### Statistical analysis

All of these analyses were carried out using GraphPad Prism from GraphPad Software (San Diego, USA). The data are presented as the mean ± SD from at least 3 separate experiments. Unless otherwise noted, the differences between the groups were analyzed using Student’s two-tailed t test when only 2 groups were compared or were assessed by one-way analysis of variance (ANOVA) when more than 2 groups were compared. *P* < 0.05 was considered statistically significant.

## Results

### MiR-7-5p is negatively correlated with dox resistance in SCLC cells and enhances doxorubicin cytotoxicity

**Fig. 1:**
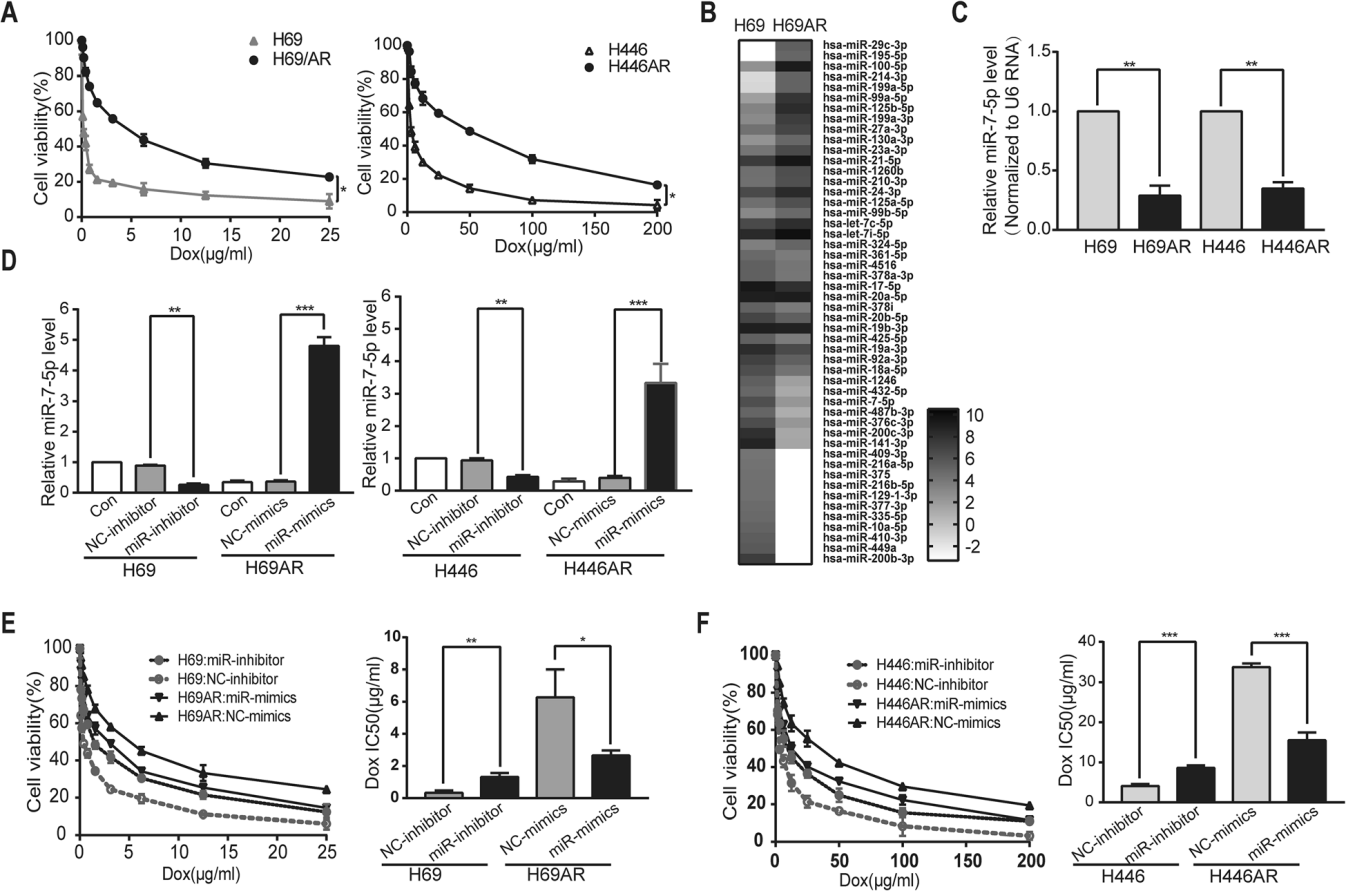
Expression of miR-7-5p in SCLC cell lines. **a** Cell viability of four SCLC cell lines (H69, H69AR, H446, and H446AR) in the presence of Dox. **b** Hierarchical clustering of differentially expressed miRNAs in H69 and H69AR cells, *black*: increased expression, *white*: decreased expression. **c** The expression of miR-7-5p in four SCLC cell lines was quantified by qRT-PCR. **d** MiR-7-5p expression in SCLC cells was determined after transfection with miR-7-5p mimic, inhibitor or negative control. **e-f** Effect of miR-7-5p expression on survival in four SCLC cell lines treated with Dox. Each cell line was treated with Dox for 24 h after transfection with miR-7-5p mimic or inhibitor, and the IC50 was calculated. Dox: Doxorubicin, Con: nontransfected, miR-mimic: miR-7-5p mimic, NC-mimic: negative control for miR-mimic, miR-inhibitor: miR-7-5p inhibitor, NC-inhibitor: negative control for miR-inhibitor. Error bars represent the mean ± SD based on three independent experiments. *P < 0.05, **P < 0.01 and ***P < 0.001

Two Dox-resistant SCLC cell lines (H69AR and H446AR) were analyzed by a CCK-8 assay. Dox-resistant SCLC cells showed significantly increased cell viability in the presence of doxorubicin compared with that of their parental cells (H69 and H446) (Fig. 1a). Agilent miRNA microarrays were used to analyze different miRNA expression profiles in H69 and H69AR cells. The cluster analysis revealed that the H69AR cells were characterized by significant changes in miRNA expression compared with H69 cells. A total of 62 miRNAs with intensity changes of more than 2-fold were differentially expressed (*P* < 0.05) in Dox-resistant H69AR cells compared with H69 cells (Fig. 1b). Among 62 miRNAs, miR-7-5p showed 4-fold decreased expression in H69AR cells compared with that in H69 cells. Furthermore, different levels of miR-7-5p expression were analyzed by qRT-PCR in H69, H69AR, H446 and H446AR cells (Fig. 1c). In addition, miR-449a, miR-200c-3p, miR-99a-5p, and miR-125b-5p levels showed significant changes in H69 and H69AR cells but not in H446 and H446AR cells (Additional file 1: Figure S1a). MiR-200b-3p, miR-141–3p, and miR-376a-3p were also differentially expressed in both H69/H69AR and H446/H446AR cells (Additional file 1: Figure S1b), but they are not involved in the regulation of Dox resistance in SCLC cells (Additional file 1: Figure S1c-d).

To confirm the association between miR-7-5p expression and Dox resistance, H69AR and H446AR cells were transfected with miR-7-5p mimic or negative control, and miR-7-5p expression was confirmed by qRT-PCR (Fig. 1d). Twenty-four hours after transfection, the cells were treated with doxorubicin, and cell viability was evaluated by CCK-8 assay. The results showed that the overexpression of miR-7-5p significantly increased the sensitivity of H69AR and H446AR cells to doxorubicin. Conversely, the miR-7-5p inhibitor induced resistance to doxorubicin in H69 and H446 cells (Fig. 1e-f). Collectively, these results showed that miR-7-5p was involved in the regulation of Dox resistance in SCLC cells.

### MiR-7-5p regulates the expression of PARP1

**Fig. 2:**
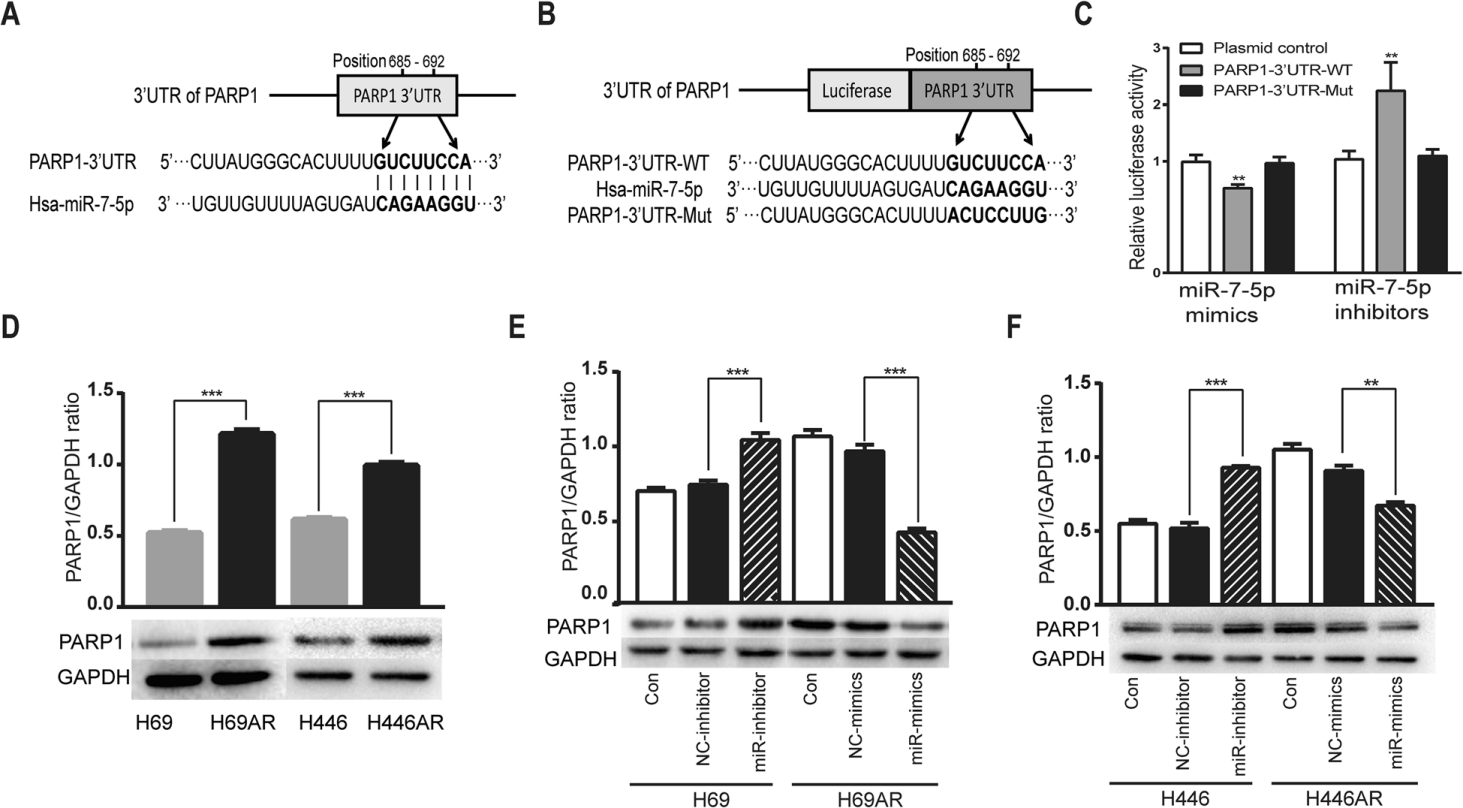
Identification of PARP1 as a target gene of miR-7-5p. **a** The binding site of miR-7-5p in the 3′ UTR of PARP1 was predicted by bioinformatic analyses. **b** Luciferase reporter constructs containing the wild-type or mutated (one point mutation) 3′ UTR of PARP1 were designed according to miR-7-5p seed sequences. **c** Dual-luciferase reporter assay. Luciferase reporter constructs and miR-7-5p mimic/inhibitor were cotransfected into HEK293 cells, and luciferase activity was detected 48 h after transfection using nontransfected cells as control. **d** The level of PARP1 protein in SCLC cells was determined by Western blot using GAPDH as a loading control. **e-f** The expression of PARP1 protein in SCLC cells with alterations in miR-7-5p levels was detected by Western blotting. Cells were transfected with miR-7-5p mimic or inhibitor, and PARP1 protein was examined by Western blot. The mean ± SD of three independent experiments (upper panel) and representative images (lower panel) are shown for each condition (**d-f**). *P < 0.05, **P < 0.01 and ***P < 0.001

To reveal the underlying molecular mechanisms by which miR-7-5p exerted its effect on Dox resistance, four prediction algorithms (DIANA, MiRcode, TargetScan, and Microrna) were utilized to predict the potential targeted genes of miR-7-5p. All the data showed that the 3′ UTR in PARP1 mRNA contains a putative binding site for miR-7-5p (Fig. 2a). To examine whether PARP1 is indeed functionally targeted by miR-7-5p, the segment of PARP1–3′ UTR-WT containing the miR-7-5p complementary site and the mutated fragment PARP1–3′ UTR-Mut were cloned into a dual luciferase reporter system (Fig. 2b). The dual-luciferase reporter assay showed that the miR-7-5p mimic significantly diminished the relative luciferase activity in cells with PARP1-WT but not in cells with PARP1-Mut (Fig. 2c). Consistently, the luciferase activity of the PARP1-WT reporter was increased when the cells were cotransfected with the miR-7-5p inhibitor. Additionally, we observed a striking inverse correlation of PARP1 levels with miR-7-5p expression. PARP1 protein levels were measured in four SCLC cells by Western blot, and the data showed that PARP1 levels were higher in the Dox-resistant cell lines (H69AR, H446AR) than in the parental cells (H69, H446) (Fig. 2d). To further confirm that miR-7-5p indeed affects the protein level of PARP1 in SCLC cells, these cells were transfected with either the miR-7-5p mimic or the inhibitor, and the levels of PARP1 were determined 48 h after transfection. These results showed that the transfection of H69AR and H446AR cells with the miR-7-5p mimic resulted in a decrease in PARP1 levels, whereas transfection with the miR-7-5p inhibitor abolished the inhibitory effect of miR-7-5p (Fig. 2e-f). Overall, these results strongly demonstrated that PARP1 is a target of miR-7-5p and is downregulated by miR-7-5p.

### Inhibition of miR-7-5p expression results in increased HR repair in dox-resistant SCLC cells

**Fig. 3:**
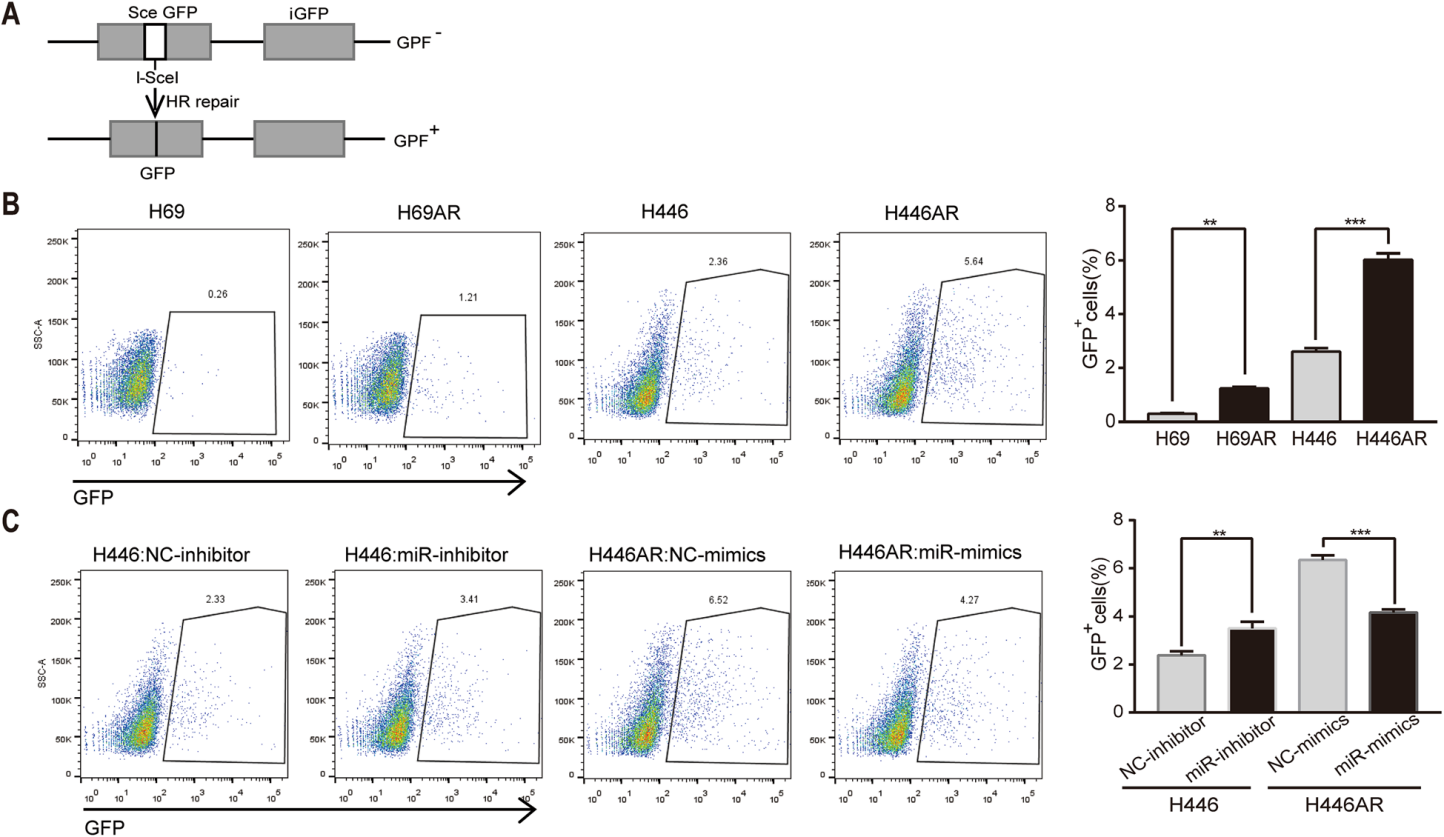
Impairment of HR repair by miR-7-5p in Dox-resistant SCLC cells**. a** Reporter construct used for the analysis of HR repair. After the introduction of DSBs by I-SceI digestion, HR repair was measured by GFP gene reconstitution in cells containing a single copy of a chromosomally integrated HR repair substrate (DR-GFP). **b** SCLC cells were cotransfected with I-SceI and pDRGFR plasmids, and then the percentage of GFP-positive cells was measured by FACS. **c** HR repair activity was impeded by miR-7-5p in SCLC cells. H446AR and H446 cells were transfected with miR-7-5p mimic or inhibitor, respectively, and the reporter construct was transiently transfected 24 h later, followed by the analysis of the GFP-positive cells by FACS. The mean ± SD of three independent experiments (right panel) and representative FACS profiles (left panel) are shown (**b-c**). *P < 0.05, **P < 0.01 and ***P < 0.001

PARP1 is a critical factor involved in the repair of DNA damage, including HR repair [15, 16]. To investigate the HR repair ability in Dox-resistant SCLC cells, we used the recombination substrate DR-GFP to monitor the repair of DSBs induced by I-SceI endonuclease [17–19]. The DR-GFP reporter system contains an upstream GFP gene with an I-SceI recognition site (SceGFP) and a downstream internal GFP repeat (iGFP). The DNA double-strand breaks are generated by expressing the I-SceI endonuclease, and repair of the cleaved I-SceI site via gene conversion-associated HR generates a functional GFP gene when the template that is repaired contains a truncated GFP fragment located downstream within the plasmid (Fig. 3a). The HR repair of I-SceI-induced DSBs can then be measured as green fluorescence using flow cytometric analysis (FACS) [20, 21]. An increased frequency of HR repair in H69AR and H446AR cells was observed compared to that in parental cells (Fig. 3b). To further confirm the effect of miR-7-5p on HR repair, H446AR cells were transfected with miR-7-5p mimic and DR-GFP reporter plasmid. Based on these experiments, we found that the miR-7-5p mimic decreased HR repair in H446AR cells. Consistent with this, H446 cells transfected with the miR-7-5p inhibitor showed an increased frequency of HR repair (Fig. 3c). Overall, these data demonstrated that miR-7-5p impaired HR repair induced by doxorubicin.

### MiR-7-5p reduces BRCA1 and Rad51 expression and disrupts HR repair induced by doxorubicin in dox-resistant SCLC cells by targeting PARP1

**Fig. 4:**
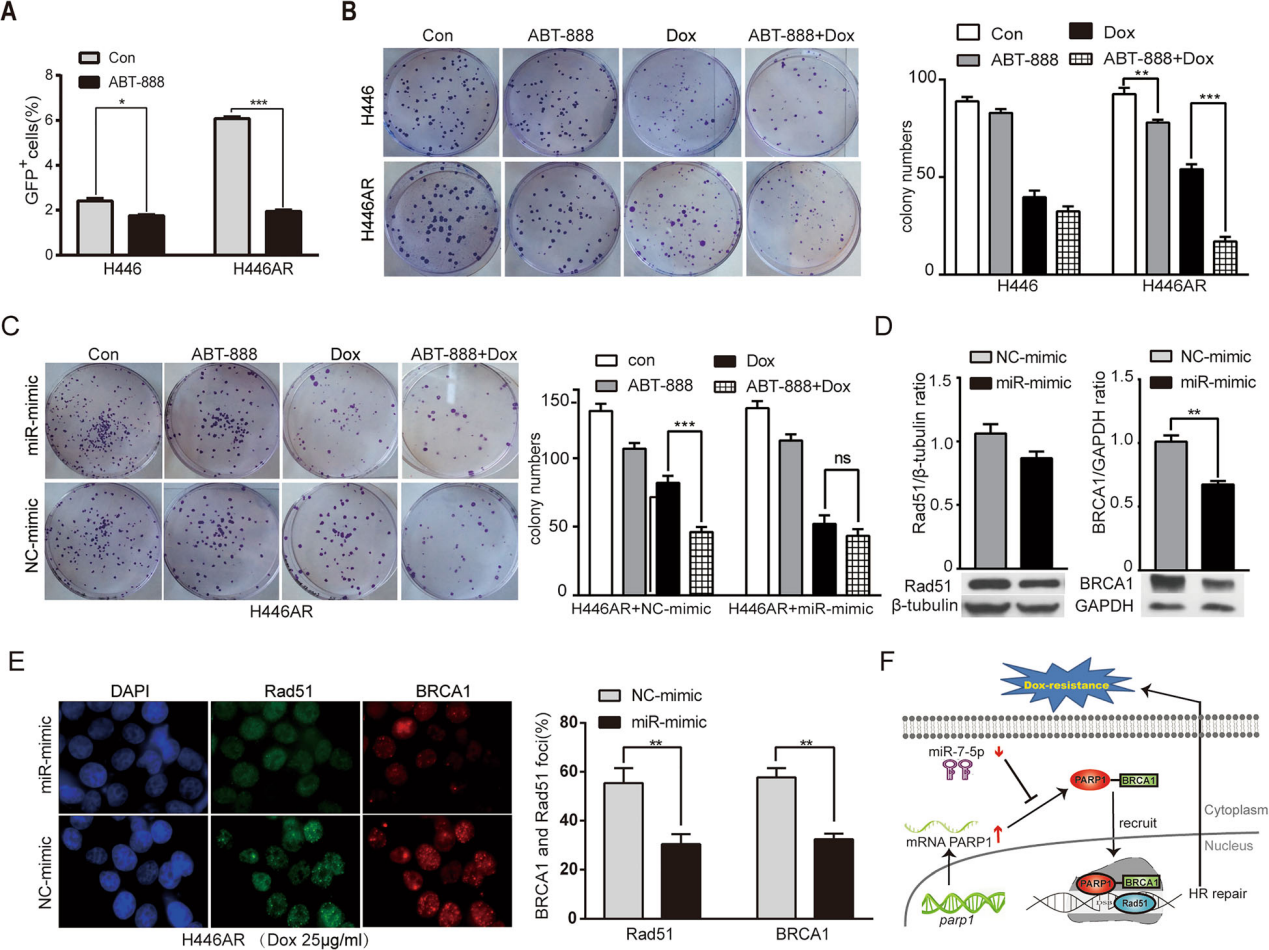
Inhibition of the HR repair pathway by miR-7-5p in Dox-resistant SCLC cells. **a** The inhibition of HR repair by PARP1 inhibitor (ABT-888) in H446 and H446AR. Cells were transfected with the reporter construct for 24 h and then treated with ABT-888 (10 μM) or control medium. HR repair activity was measured 24 h after the addition of ABT-888 by FACS. **b** Colony formation assays. H446A and H446AR cells were treated with ABT-888 alone, doxorubicin alone or combination of both for 14 d, and the quantification of the number of colonies was performed for each cell line. **c** H446AR cells were transfected with miR-7-5p mimic or NC-mimic, and the quantification of the number of colonies was performed in each group. **d** The expression of Dox-induced Rad51 and BRCA1 protein in H69AR cells with upregulated miR-7-5p levels. H446AR cells transfected with a miR-7-5p mimic were treated with 25 μg/ml doxorubicin for 24 h, and the Rad51 and BRCA1 protein levels were examined by Western blot. **e** Quantification of Dox-induced BRCA1 and Rad51 foci formation in H446AR cells. H446AR cells transfected with a miR-7-5p mimic were treated with 25 μg/ml doxorubicin for 24 h, and then the percentages of BRCA1 and Rad51 foci were determined by immunofluorescent staining. The mean ± SD of three independent experiments (right panel) and representative images (left panel) are shown (**b-c**). **f** A working model of miR-7-5p function in Dox-resistant SCLC. The downregulation of miR-7-5p promoted HR repair in DNA by targeting PARP1 and conferring chemo-resistance against doxorubicin. *P < 0.05, **P < 0.01 and ***P < 0.0

Since PARP1 is a target of miR-7-5p and plays a crucial role in the HR repair pathway, we next investigated the role of the PARP1 inhibitor ABT-888 (Veliparib) in HR repair in H446 and H446AR cells. Our results showed that ABT-888 caused a more significant decrease in HR activity in H446AR cells than in H446 cells (Fig. 4a). In addition, colony formation assays were used to determine the role of PARP1 in Dox-induced HR repair and cell growth. The results demonstrated that the number of colonies were dramatically reduced by PARP1 alone or in combination with the PARP1 inhibitor doxorubicin in H446AR cells, while no significant change was observed in H446 cells (Fig. 4b). To further elucidate the role of the miR-7-5p/PARP1 axis in Dox-resistant cells, H446AR cells were transfected with miR-7-5p mimic and scrambled miRNA (NC-mimic). Colony formation assays demonstrated that H446AR cells were resensitized to doxorubicin after transfection with the miR-7-5p mimic, while cotreatment with the PARP1 inhibitor and doxorubicin did not result in synergistic effects (Fig. 4c). Collectively, these data suggested that the downregulation of miR-7-5p promoted PARP1 expression, resulting in enhanced HR repair that confers Dox resistance. Furthermore, the expression of the HR-associated markers Rad51 and BRCA1 was analyzed in H446AR cells after treatment with doxorubicin. Western blotting and immunofluorescent double staining demonstrated that Rad51 and BRCA1 were both inhibited by miR-7-5p mimic transfection but not by miRNA NC-mimic transfection, indicating that HR repair induced by doxorubicin was impaired by miR-7-5p in H446AR cells (Fig. 4d-e). Overall, our findings indicated that miR-7-5p downregulated the expression of Rad51 and BRCA1 and resensitized SCLC cells to doxorubicin by targeting PARP1 mRNA.

## Discussion

Advanced SCLC is an aggressive disease associated with major morbidity and mortality [22]. Chemo-resistance is a major obstacle for SCLC treatment. Therefore, there is a great need to clarify the mechanisms of chemo-resistance underlying the clinical behavior of SCLC. Recently, an increasing number of studies has demonstrated that the ectopic expression of miRNAs may be involved in the acquisition of tumor cell resistance to chemotherapy by altering the efficacy of DNA damage repair [11, 12]. Let-7 was reported to regulate BRCA1 and Rad51 expression and subsequently enhance DNA repair, which results in cisplatin resistance [23]. However, the molecular mechanisms of miRNA involved in DNA damage repair and chemo-resistance in SCLC cells are still unclear. In this study, we provided data indicating the importance of miRNA expression in the acquisition of SCLC cell resistance to doxorubicin. This was evidenced by the pronounced alteration in the expression of miRNA genes in Dox-resistant cells compared with parental cells. MiRNA profiling revealed that miR-7-5p was dramatically downregulated in Dox-resistant SCLC cells, which was validated by qRT-PCR. MiR-7-5p has been reported to be a tumor suppressor due to its ability to suppress cell growth and induce cell apoptosis [13, 24–26]. Liu and colleagues demonstrated that miR-7-5p mediated SCLC chemo-resistance by repressing the expression of ABCC1 gene, which is a typical ABC transporter [27]. Guo and colleagues reported that the expression of inwardly rectifying potassium channel Kir2.1 (KCNJ2) was regulated by miR-7-5p that modulated multiple drug resistance in SCLC [28]. However, the functional role of miR-7-5p in regulating DNA repair during chemo-resistance has not yet been elucidated. Our results demonstrated that miR-7-5p was involved in the chemo-resistance of SCLC cells against doxorubicin via DNA repair pathway.

To explore the potential mechanisms underlying the involvement of miR-7-5p in Dox-resistance in SCLC cells, we utilized bioinformatics analysis and dual-luciferase reporter system to predict the genetic targets of miR-7-5p. The results showed that miR-7-5p directly targeted PARP1 and suppressed the expression of PARP1 in Dox-resistant SCLC cells. PARP1 is a nuclear enzyme that plays a critical role in many biological processes, including DNA repair and gene transcription [29, 30]. PARP1 is activated by DNA damage and catalyzes the polymerization of ADP-ribose units, which results in the rapid recruitment of DNA repair proteins to sites of DNA damage [31]. There are two major pathways for DSB repair: nonhomologous end joining (NHEJ) and homologous recombination (HR). PARP1 is the key determinant of the HR repair pathway, and HR repair is crucial for maintaining genomic integrity and survival in response to chemotherapy [32]. Our results further showed that HR repair was increased in Dox-resistant SCLC cell lines compared to that in parental cells. Considering the role of PARP1 in HR repair, we can speculate that the downregulation of miR-7-5p promotes PARP1 expression, which in turn enhances HR repair and causes chemo-resistance.

BRCA1 and Rad51 are key factors in HR repair. Previous studies have demonstrated that PARP1 is important for BRCA1 recruitment to DSBs and that PARP1 depletion results in the loss of BRCA1 foci formation [33, 34]. PARP1 modulates the initial steps of DSB repair that involve the binding of Rad51 to DNA to stimulate strand exchange during HR. It has been reported that PARP1 chemical inhibitors or siRNAs targeted to PARP1 can inhibit HR by suppressing the expression of BRCA1 and Rad51 [35]. Consistent with this observation, our results demonstrated that miR-7-5p inhibited the expression of BRCA1 and Rad51 that was induced by doxorubicin in Dox-resistant SCLC cells by suppressing PARP1 expression (Fig. 4f). Our findings suggested that PARP1-mediated HR activity promoted cell survival in doxorubicin-resistant SCLC cells and that miR-7-5p could be used as a sensitizer to overcome chemo-resistance.

## Conclusion

The current study shows for the first time the correlation between miR-7-5p expression and HR repair in SCLC cells Dox resistance. Further investigation showed that PARP1-mediated HR activity was impaired by miR-7-5p. Considering the indispensable function of PARP1 during HR repair, our findings suggest that miR-7-5p may serve as a potential biomarker of chemo-resistance and may be used as a new therapeutic approach to overcome chemo-resistance.

## Additional file

Additional file 1: **Figure S1.** Expression of miR-449a, miR-200c-3p, miR-99a-5p, miR-125b-5p, miR-200b-3p, miR-141–3p, and miR-376a-3p in SCLC cell lines. **a-b** The expression of miR-449a, miR-200c-3p, miR-99a-5p, miR-125b-5p, miR-200b-3p, miR-141–3p, and miR-376a-3p in four SCLC cell lines was quantified by qRT-PCR. **c-d** Effect of miR-200b-3p and miR-141–3p expression on survival in four SCLC cell lines treated with Dox. Each cell line was treated with Dox for 24 h after transfection with the mimic or inhibitor, and the IC50 was calculated. Dox: Doxorubicin, miR-mimic: miR-200b-3p or miR-141–3p mimic, NC-mimic: negative control for the miR-mimic, miR-inhibitor: miR-200b-3p or miR-141–3p inhibitor, NC-inhibitor: negative control for the miR-inhibitor. Error bars represent the mean ± SD of three independent experiments. *P < 0.05, **P < 0.01 and ***P < 0.001. (TIF 1124 kb)

## Data Availability

The datasets supporting the conclusions of the current study are available from the corresponding author on reasonable request. Please contact corresponding author, if you want to request the dataset.

## Abbreviations

3′-UTR: 3′-untranslated region
DDR: DNA damage response
Dox: Doxorubicin
DSBs: Double-strand breaks
HR: Homologous recombination
PARP1: Poly ADP-ribose polymerase 1
SCLC: Small cell lung cancer
