# Supplementary figures and images for: MiR-7-5p-mediated downregulation of PARP1 impacts DNA homologous recombination repair and resistance to doxorubicin in small cell lung cancer
Source: BMC Cancer. 2019 Jun 18;19:602. doi: 10.1186/s12885-019-5798-7 (PMC6582543; doi:10.1186/s12885-019-5798-7)

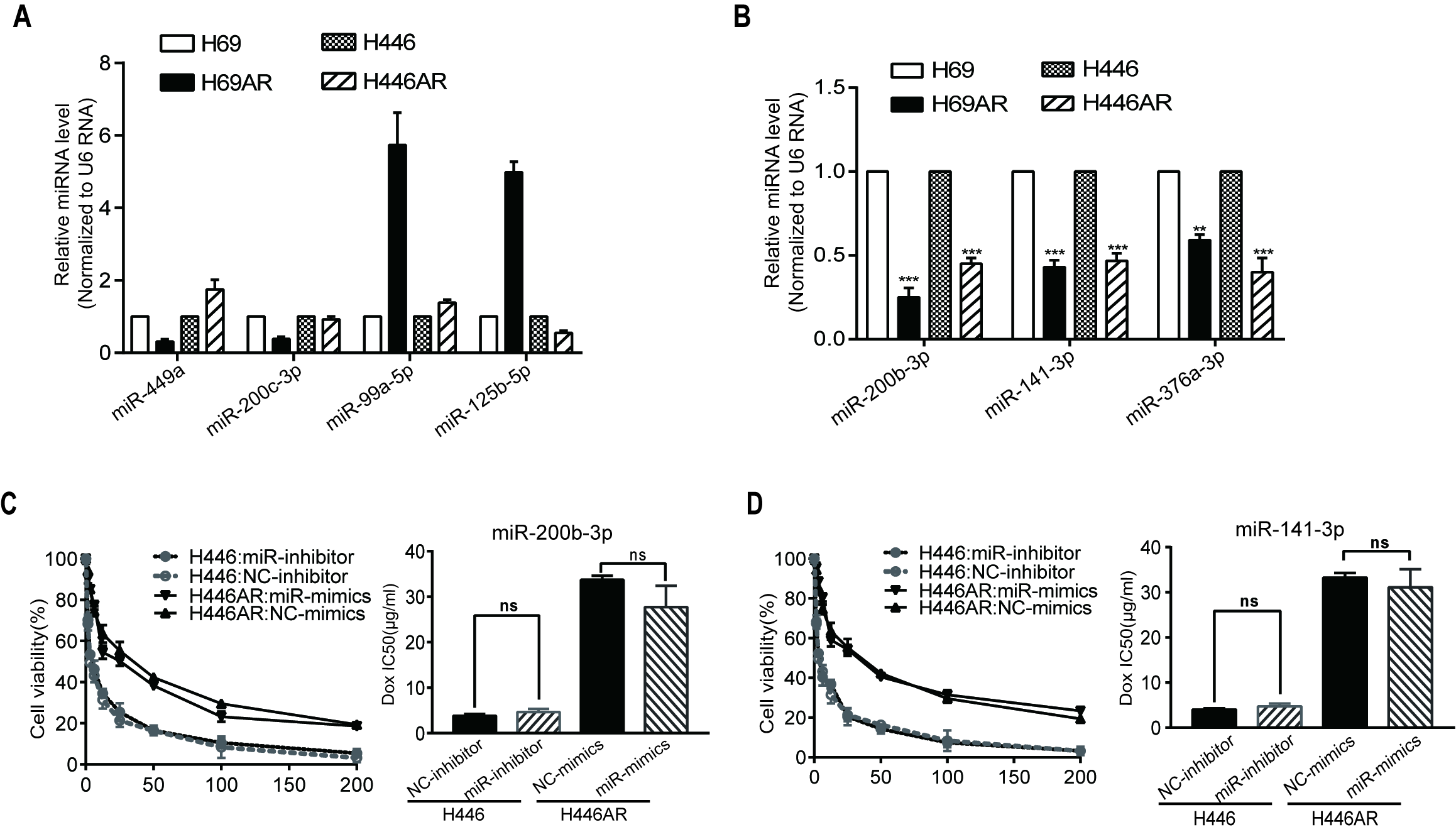

Supplement: Supplementary file 1 — Figure S1. Expression of miR-449a, miR-200c-3p, miR-99a-5p, miR-125b-5p, miR-200b-3p, miR-141–3p, and miR-376a-3p in SCLC cell lines. a-b The expression of miR-449a, miR-200c-3p, miR-99a-5p, miR-125b-5p, miR-200b-3p, miR-141–3p, and miR-376a-3p in four SCLC cell lines was quantified by qRT-PCR. c-d Effect of miR-200b-3p and miR-141–3p expression on survival in four SCLC cell lines treated with Dox. Each cell line was treated with Dox for 24 h after transfection with the mimic or inhibitor, and the IC50 was calculated. Dox: Doxorubicin, miR-mimic: miR-200b-3p or miR-141–3p mimic, NC-mimic: negative control for the miR-mimic, miR-inhibitor: miR-200b-3p or miR-141–3p inhibitor, NC-inhibitor: negative control for the miR-inhibitor. Error bars represent the mean ± SD of three independent experiments. *P < 0.05, **P < 0.01 and ***P < 0.001. (TIF 1124 kb) [file 12885_2019_5798_MOESM1_ESM.tif]
